# Clinical and Epidemiological Profiles of Severe Malaria in Children from Delhi, India

**DOI:** 10.3329/jhpn.v30i1.11291

**Published:** 2012-03

**Authors:** Jaya Shankar Kaushik, Sunil Gomber, Pooja Dewan

**Affiliations:** Department of Pediatrics, University College of Medical Sciences and Guru Teg Bahadur Hospital, Delhi 110095, India

**Keywords:** Child, Malaria, *Plasmodium vivax*, Prospective studies, India

## Abstract

*Plasmodium vivax* is traditionally known to cause benign tertian malaria, although recent reports suggest that *P. vivax* can also cause severe life-threatening disease analogous to severe infection due to *P. falciparum*. There are limited published data on the clinical and epidemiological profiles of children suffering from ‘severe malaria’ in an urban setting of India. To assess the clinical and epidemiological profiles of children with severe malaria, a prospective study was carried out during June 2008–December 2008 in the Department of Pediatrics, Guru Teg Bahadur Hospital, a tertiary hospital located in East Delhi, India. Data on children aged ≤12 years, diagnosed with severe malaria, were analyzed for their demographic, clinical and laboratory parameters. All patients were categorized and treated as per the guidelines of the World Health Organization. In total, 1,680 children were screened for malaria at the paediatric outpatient and casualty facilities of the hospital. Thirty-eight children tested positive for malaria on peripheral smear examination (2.26% slide positivity rate). Of these, 27 (71%) were admitted and categorized as severe malaria as per the definition of the WHO while another 11 (29%) received treatment on outpatient basis. Most (24/27; 88.8%) cases of severe malaria (n=27) were infected with *P. vivax*. Among the cases of severe malaria caused by *Plasmodium vivax* (n=24), 12 (50%) presented with altered sensorium (cerebral malaria), seven (29.1%) had severe anaemia (haemoglobin <5 g/dL), and 17 (70.8%) had thrombocytopaenia, of which two had spontaneous bleeding (epistaxis). Cases of severe vivax malaria are clinically indistinguishable from severe falciparum malaria. Our study demonstrated that majority (88.8%) of severe malaria cases in children from Delhi and adjoining districts of Uttar Pradesh were due to *P. vivax*-associated infection. *P. vivax* should, thus, be regarded as an important causative agent for severe malaria in children.

## INTRODUCTION

Malaria continues to be a major public-health problem in most tropical countries. As per the World Malaria Report 2009, there were 1.53 million confirmed cases of malaria from India, of which 0.77 million were due to *Plasmodium falciparum* (1). Jharkhand, Orrisa, Chhattisgarh, Madhya Pradesh, and West Bengal contributed to half of the total malaria cases in India (2). Urban malaria constitutes 15% of the burden of the disease in India (2). Mortality is very high in untreated severe forms of malaria. Cerebral malaria and anaemia are the two most important manifestations of severe malaria in children.

Traditionally, severe malaria has been associated with infection due to *P. falciparum*. Recent studies from South-East Asia have highlighted *P. vivax* as a major cause of morbidity and mortality in infants and children (3,4). Data on the clinical and epidemiological profiles of children with severe malaria from urban centres are limited. Hence, we analyzed the profile of severe malaria in children who were admitted to a tertiary centre located in East Delhi, catering to population from Delhi and adjoining districts of Uttar Pradesh, India.

## MATERIALS AND METHODS

### Study design and setting

A prospective study was conducted during June–December 2008 in a tertiary-care hospital located in East Delhi, which caters to population from Delhi and nearby districts (Ghaziabad, Meerut, Baghpat, Kannoj, and Bulandshahr of Uttar Pradesh).

### Enrollment

The study was conducted among children aged less than or equal to 12 years. Children with a short duration (<3 days) of fever (temperature >38 °C) without any localized symptoms were screened from the paediatric outpatient and casualty.

### Procedure

The diagnosis of malaria, including identification of species, was established by examination of thick and thin smears of peripheral blood. Slides were prepared, stained with Giemsa, and examined under an oil immersion microscope. At least 100 high-power fields (40X) of the thick smears were examined to rule out malaria. Malarial antigen test (optiMAL) and real-time polymerase chain reaction (PCR) could not be done due to their non-availability at our centre, although these are considered better sensitive methods for the detection of *Plasmodium* species.

The demographic profile, clinical features, and clinical course of illness were recorded. A baseline haemogram with platelet count, blood sugar, serum creatinine, prothrombin time, partial thromboplastin time, blood lactate, pH estimation, urine examination, and chest skiagram were performed in all the cases. Investigations, such as cerebrospinal fluid examination, urine culture, blood culture, neuroimaging, ultrasonography of abdomen, and liver function tests (serum bilirubin and serum aminotransferases), were conducted where deemed essential.

### Diagnosis and treatment protocol

Based on the clinical and laboratory parameters, children who were categorized as severe malaria as per the guidelines of the World Health Organization (WHO) were included in the study (5). Children diagnosed with severe malaria were treated with either intravenous quinine (20 mg/kg, followed by 10 mg/kg tid for 7 days) or intravenous artesunate (2.4 mg/kg stat, followed by 2.4 mg/kg after 12 hours, then once a day for 7 days). When the patient was able to accept orally, s/he was shifted to either oral quinine or oral artesunate accompanied with clindamycin, 15 mg/kg/day, orally, for seven days (5). Children with uncomplicated malaria were treated with artemisinin combination therapy (ACT) or with oral chloroquine (10 mg/kg stat, followed by 5 mg/kg after six hours, 24 hours, and 48 hours) in the case of *P. vivax*. Primaquine was given in the dose of 0.25 mg/kg/day for 14 days in the case of *P. vivax* and a single dose of 0.75 mg/kg for *P. falciparum*.

### Ethical aspects

The study protocol was fully explained to the parents/guardians, and informed written consent was obtained before recruitment.

## RESULTS

In total,1,680 children were screened for malarial infection in our centre during the study period. Of these, 38 children tested positive for malaria on peripheral smear examination, yielding a slide positivity rate of 2.26%. Of these, 35 (92.1%) were caused by *P. vivax*, and the remaining three cases were caused by *P. falciparum* (7.9%). Twenty-seven cases were categorized as severe malaria as per the guidelines of the WHO and were admitted for treatment. The remaining 11 children (all having *P. vivax*-associated malaria) received treatment as outpatients.

Among the children with severe malaria, *P. vivax* was documented in 24 (88.8%) and *P. falciparum* in three cases (11.1%). The clinical and laboratory profiles of children with severe vivax malaria are presented in the table. The age of the patients with severe vivax malaria (n=24) ranged from eight months to 12 years; 18 (75%) of them were male. Fever was the chief presenting complaint in all the patients (100%), with a median duration of six (range 2-9) days. Other presenting complaints of children with severe vivax malaria were: repeated vomiting (n=18; 75%), altered sensorium/encephalopathy (n=12; 50%), seizures (n=8; 33.3%), and epistaxis (n=2; 8.3%). The clinical signs among these patients included hepatomegaly (24; 100%), splenomegaly (n=22; 91.6%), pallor (n=20; 83.3%), icterus (n=2; 8.3%), and respiratory distress (n=2; 8.3%). Thirteen (54%) children had more than one manifestation of severe malaria. Of the 12 children with encephalopathy, eight had multiple convulsions, two had severe anaemia requiring blood transfusion, and one had spontaneous bleeding. Lumbar puncture was done in 10 of the 12 children presenting with altered sensorium, which were all acellular with normal cerebrospinal fluid biochemical parameters.

Seven (29.1%) patients with severe anaemia (haemoglobin <5 g/dL) required blood transfusion. Thrombocytopenia (platelet <1.5 lakh/dL) was observed in 17 (70.8%) children while acute renal failure (serum creatinine >1.5 mg/dL) was seen in two (8.3%) cases. Of the 24 children with severe vivax malaria, two also had lobar pneumonia requiring intravenous antibiotics (ceftriaxone and cloxacillin). Blood and urine cultures were sterile in all the children.

**Table.** Clinical and laboratory features of severe vivax malaria

Severe vivax malaria	No. of patients (n=24)	%
Clinical features		
Impaired consciousness	12	50
Multiple convulsions	8	33.3
Respiratory distress	2	8.3
Circulatory collapse or shock	2	8.3
Abnormal spontaneous bleeding	2	8.3
Laboratory features		
Severe anaemia (Hb <5 g/dL)	7	29.1
Thrombocytopaenia	17	70.8
Renal impairment	2	8.3

All the patients with severe vivax malaria responded to intravenous quinine. Two patients with acute renal failure were started on intravenous artesunate, followed by oral therapy once the patient started accepting orally, for seven days. All the cases of uncomplicated malaria were treated with oral chloroquine as none of the patients could afford ACT. The outcome of the patients was favourable in all the cases with severe vivax malaria. However, one child diagnosed as cerebral malaria due to *P. falciparum*-associated infection died within a few hours of hospitalization.

## DISCUSSION

The present study showed a surge of cases of severe malaria caused by *P. vivax* among children residing in East Delhi and neighbouring districts of Uttar Pradesh during the study period. Clinical features of severe malaria caused by *P. vivax* were similar to those caused by *P. falciparum,* which included altered sensorium, severe anaemia, and thrombocytopaenia. These data on severe vivax malaria are in line with those reported from Papau New Guinea and Indonesia (3,4).

Manifestations of severe vivax malaria include severe anaemia, respiratory distress, coma, malnutrition, splenic rupture, thrombocytopaenia, and acute renal failure and shock. Features of severe vivax malaria in our study were cerebral malaria, severe anaemia, and thrombocytopaenia. There has been a recent surge of cases of cerebral malaria caused by *P. vivax* (6,7). Sequestration of infected red blood cells in cerebral vessels is a feature of cerebral malaria. Results of a study by Kochar *et al.* showed that vivax malaria can have both sequestration-related and non-sequestration-related complications of severe malaria, including cerebral malaria (8). Profound thrombocytopaenia is uncommon in malaria due to *P. vivax*, although it is well-documented in *P. falciparum*-associated malaria (9). It could be a result of direct lysis of platelets by *P. vivax* or by immunological destruction by platelet-associated IgG antibody. We observed thrombocytopaenia in 74.1% of the malarial children, a significantly high proportion, although similar paediatric cases have been reported earlier (10).

Co-morbidities are common in endemic areas of malaria and could contribute significantly to mortality among cases of severe vivax malaria (11). Our patients of severe vivax malaria had co-morbidity of severe anaemia (29.1%), pneumonia (8.3%), acute renal failure (8.3%), and respiratory distress (8.3%). Despite the high rates of co-morbidities and severe manifestation of vivax malaria, the mortali-ty rate in our study was nil. The mortality rate in severe vivax malaria ranged from 0.8% to 1.6% (12). This implies that timely initiation of treatment with recommended antimalarial therapy, along with other supportive management, could lead to the low-mortality outcome.

There has been an epidemiological change in the pattern of severe malaria in our region where *P. falciparum* was mostly considered the causative agent (13). A possible explanation for this emerging trend may be due to increase in migrant population in Delhi owing to more job opportunities and development of resettlement colonies bringing in a fresh cohort of children with different immune status and, hence, susceptibility to *Plasmodium* infection. Also, the increase in construction activities in and around Delhi has led to an increased breeding of mosquitoes and, hence, a rise in mosquito-borne diseases, including malaria and dengue. Since *P. vivax* was considered less virulent, surveillance reports in the past had primarily focused on *P. falciparum*. This could also explain the limited data available on the clinical features of severe malaria due to *P. vivax*. The emergence of severe malaria due to *P. vivax* as observed in our study might have important implications for the community with respect to treatment and prevention strategies.

The emergence of severe malaria caused by *P. vivax* could have significant implications in planning the malaria-control programmes for the community. The widespread use of ACT for severe malaria as per the guidelines of the WHO could result in the emergence of resistant parasite strains. It may be rational to continue the use of chloroquine for malaria due to *P. vivax* in chloroquine-sensitive areas. In places where both *P. vivax* and *P. falciparum* co-exist, measures need to be equally targeted to *P. vivax* to decrease morbidity and mortality due to severe malaria.

The present study highlighted the change in the epidemiology of childhood severe malaria in the urban setting of North India. However, annual reporting with multicentric design of prospective studies is required to establish and confirm this trend.
